# Reduced GABAergic transmission in the ventrobasal thalamus contributes to thermal hyperalgesia in chronic inflammatory pain

**DOI:** 10.1038/srep41439

**Published:** 2017-02-02

**Authors:** Chan Zhang, Rong-Xiang Chen, Yu Zhang, Jie Wang, Feng-Yu Liu, Jie Cai, Fei-Fei Liao, Fu-Qiang Xu, Ming Yi, You Wan

**Affiliations:** 1 Neuroscience Research Institute and Department of Neurobiology, School of Basic Medical Sciences, Peking University, Beijing 100191, P.R. China; 2 Department of Neurology, The First Affiliated Hospital of Zhengzhou University, Zhengzhou, Henan 450052, P.R. China; 3 Research Center for Medicine and Biology, Zunyi Medical University, Zunyi 563000, P.R. China; 4 Key Laboratory of Magnetic Resonance in Biological Systems, State Key Laboratory of Magnetic Resonance and Atomic and Molecular Physics, Wuhan Institute of Physics and Mathematics, Chinese Academy of Sciences, Wuhan 430071, P.R. China; 5 Key Laboratory for Neuroscience, Ministry of Education/National Health and Family Planning Commission, Beijing 100191, P.R. China

## Abstract

The ventrobasal (VB) thalamus is innervated by GABAergic afferents from the thalamic reticular nucleus (TRN) and participates in nociception. But how the TRN-VB pathway regulates pain is not fully understood. In the present study, we reported decreased extracellular GABA levels in the VB of rats with CFA-induced chronic inflammatory pain, measured by microdialysis with HPLC analysis. *In vitro* whole-cell patch-clamp recording showed decreased amplitudes of tonic currents, increased frequencies of mIPSCs, and increased paired-pulse ratios in thalamic slices from chronic inflammatory rats (7 days). Microinjection of the GABA_A_R agonist muscimol and optogenetic activation of the TRN-VB pathway relieved thermal hyperalgesia in chronic inflammatory pain. By contrast, microinjecting the extrasynaptic GABA_A_R agonist THIP or selective knockout of synaptic GABA_A_R γ2 subunits aggravated thermal hyperalgesia in the chronic stage of inflammatory pain. Our findings indicate that reduced GABAergic transmission in the VB contributes to thermal hyperalgesia in chronic inflammatory pain, which could be a synaptic target for pharmacotherapy.

The ventrobasal thalamus (VB), including the ventral posterolateral (VPL) and the ventral posteromedial (VPM) nuclei, gates nociceptive information to the cerebral cortex. Both VPM and VPL are subject to GABAergic modulation from the thalamic reticular nucleus (TRN)^1^,^2^,^3^,^4^,^5^. GABAergic modulation in the VB is preferentially mediated by GABA type A receptors (GABA_A_Rs), despite the involvement of GABA type B receptors^4^,^6^,^7^. GABA_A_Rs are pentametric ligand-gated ion channels composed of two α (1–6), two β (1–3) subunits and one special subunit δ, γ (1–3), θ, ε, π, ρ (1–3)^8^,^9^. In the thalamus, GABA_A_Rs are mainly composed of α1, α4, β2, δ and γ2 subunits^10^. GABA released from presynaptic vesicles as a neurotransmitter binds to synaptic GABA_A_Rs (α1β2γ2) and mediates the transient or phasic inhibition; whereas ambient GABA binds to extra-synaptic GABA_A_Rs (α4β2δ) to mediate the persistent or tonic inhibition^6^,^11^. Functionally, the diversity of GABAergic inhibition in the thalamus participates in a variety of physiological and pathological conditions, such as attention, sleep, absence epilepsy and tinnitus^5^.

Anatomical, biochemical and electrophysiological changes occur in the VB under inflammatory and other chronic pain conditions^12^,^13^,^14^,^15^,^16^. Patients with neuropathic pain display significant volume loss of the somatosensory thalamus associated with decreased TRN activities^13^. However, the exact role of thalamic GABAergic transmission in chronic pain has not been sufficiently investigated. Based on previous findings, we hypothesize that chronic pain is accompanied by altered thalamic GABAergic transmission, which contributes to the thermal hyperalgesia commonly seen in this disorder. In the present study, we examined this hypothesis with a combinative application of microdialysis, electrophysiological recording, behavioral testing, and pharmacological and optogenetic manipulations in rats or mice with complete Freund’s adjuvant (CFA)-induced chronic inflammatory pain.

## Results

### Decreased extracellular GABA levels in the VB of rats with CFA-induced chronic inflammatory pain

**Figure 1:**
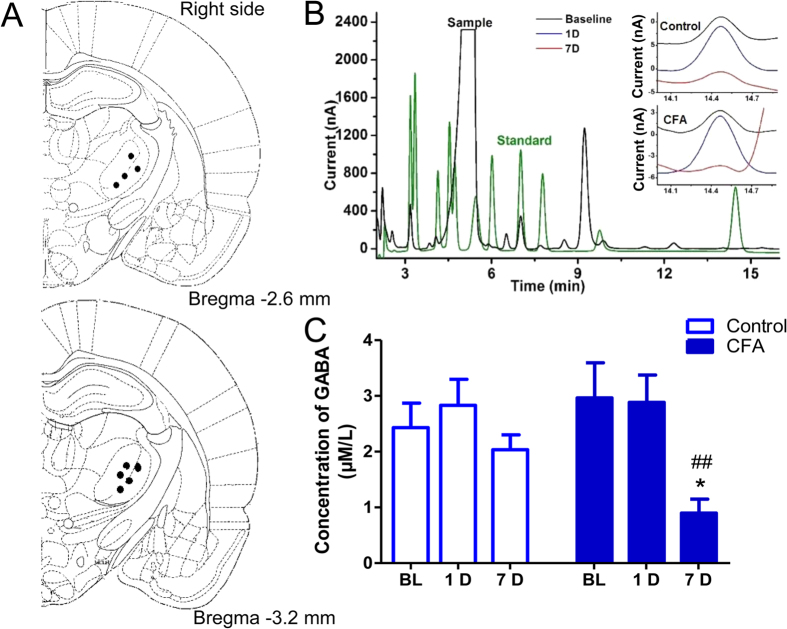
Decreased extracellular GABA levels in the VB of rats with chronic inflammatory pain. (**A**) Histological confirmation of microdialysis probe tips. Brain slices at the level of 2.6 mm/3.2 mm caudal to the Bregma showed the probe tip in the right VB in the control and CFA groups. (**B**) Brain perfusate samples (black) and internal standards (green) were detected with high-performance liquid chromatography (HPLC). GABA was eluted at ~14.5 min. Chromatograms of OPA derivatives of GABA from a control rat (top) and an inflammatory rat (bottom) were shown at different time points: Baseline (black line); day 1 (blue line) and day 7 (red line). (**C**) Saline injection did not affect GABA levels in the VB, whereas the level of GABA decreased 7 days after CFA injection (CFA-7D). Error bars indicated SEMs, *P < 0.05, two-way ANOVA with Bonferroni post-test. BL: baseline.

We first examined the level of extracellular GABA *in vivo* in the VB of rats with CFA-induced inflammatory pain using microdialysis at the perfusion rate 0.1 μl/min (Fig. 1A). Figure 1B showed the chromatograms of O-phthalaldehyde (OPA) derivatives of GABA from the CFA and control groups at different time points (baseline, and days 1 and 7 after CFA/NS injection). GABA levels in the VB showed no obvious changes in rats in the control group, but were significantly lower in the CFA group on day 7 (CFA-7D) [Fig. 1C, F(2, 24) = 4.765, p = 0.018]. These results indicate decreased extracellular GABA levels in the VB of rats with chronic inflammatory pain.

### Altered thalamic GABAergic transmission in rats with CFA-induced chronic inflammatory pain

**Figure 2:**
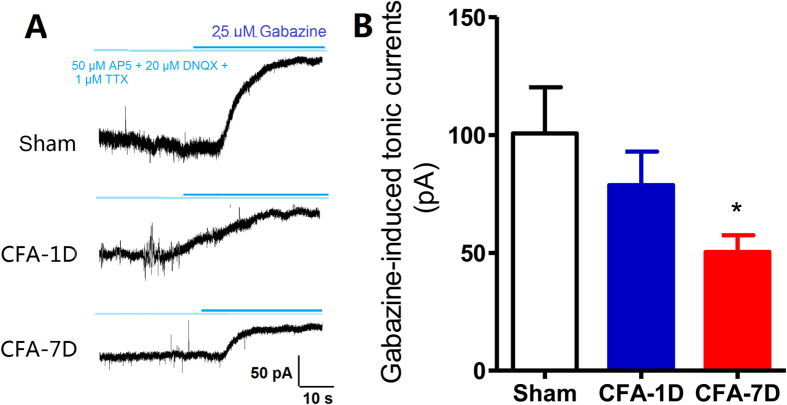
Decreased tonic inhibition of extra-synaptic GABA_A_Rs in the VB in CFA-induced chronic inflammatory pain rats (CFA-7D). Representative traces of tonic inhibition recordings from thalamic relay neurons in the control (n = 8 cells), CFA-1D (n = 10 cells) and CFA-7D (n = 10 cells) groups after inhibiting ionotropic glutamatergic components (AP5 50 μM, DNQX 20 μM) and sodium currents (TTX 1 μM). Gabazine (25 μM), a competitive GABA_A_Rs antagonist, produced a positive shift and blocked both phasic and tonic currents. (**B**) Maximal current changes after gabazine (25 μM) application measured as the shift of GABAergic tonic inhibition (∆IPSC_tonic_ = IPSC_after_ − IPSC_before_). The histogram of tonic inhibitory currents showed a reduction of tonic inhibitory currents in the CFA-7D, but not the CFA-1D groups. Error bars indicated SEMs, *P < 0.05 compared with the control group, one-way ANOVA with Tukey post*-*test.

To explore the electrophysiological dynamics of thalamic GABAergic transmission in chronic inflammatory pain, we performed whole-cell patch-clamping in the thalamic slice contralateral to the CFA injection side. With a −70 mV holding potential, we first observed tonic currents of extra-synaptic GABA_A_Rs under the blockage of the GABA_A_R antagonist gabazine (25 μM) (Fig. 2A). As shown in Fig. 2B, amplitudes of tonic currents in the CFA-1D group were similar to those in the control group [100 ± 19.50 pA *vs.* 78.75 ± 14.20 pA, F(2, 25) = 1.22, p = 0.281], but decreased in the CFA-7D group [50.38 ± 7.13 pA, F(2, 25) = 5.63, p = 0.026]. No significant differences were observed between control-1D and control-7D rats and the data were pooled into the control group (control-1D, 111.1 ± 31.19 pA; control-7D, 90.25 ± 27.02 pA, p = 0.63). These results, consistent with decreased extracellular levels of GABA, indicate decreased tonic currents of extra-synaptic GABA_A_Rs in the VB in chronic inflammatory pain rats.

**Figure 3:**
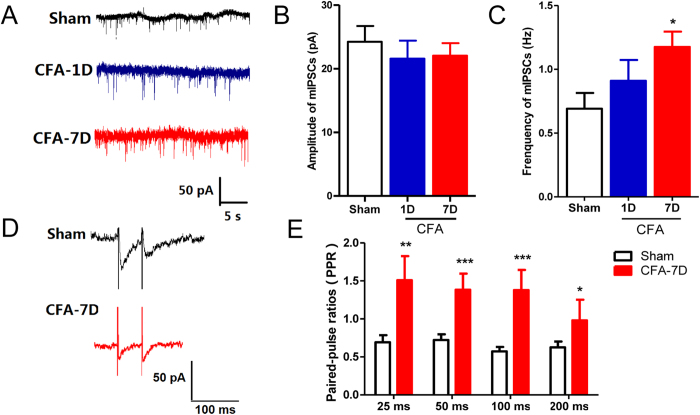
GABAergic transmission in the VB in chronic inflammatory pain revealed by whole-cell patch clamp recording. (**A**) Representative synaptic GABA_A_Rs-mediated mIPSC traces in thalamic relay neurons in the control (normal saline, n = 16 cells), CFA-1D (n = 14 cells) and CFA-7D (n = 20 cells) groups. (**B**) Similar amplitudes of mIPSCs in three groups. (**C**) Compared with the control group, the frequency of mIPSCs remained unchanged in the CFA-1D group, but significantly increased in the CFA-7D group. (**D**) Representative IPSC pairs evoked by paired-pulse stimulations with a 50-ms interval in the control (n = 7 cells) and CFA-7D (chronic inflammatory pain, n = 9 cells) groups. (**E**) Increased paired-pulse ratios (PPRs = IPSC_2nd_/IPSC_1st_) induced by paired-pulse stimulation with 25-, 50-, 100- or 200-ms intervals in the CFA-7D group. Error bars indicated SEMs. *P < 0.05, **P < 0.01, one-way ANOVA with Tukey post-test for mIPSCs and two-way ANOVA with Bonferroni post-test for PPR analysis.

We further examined other electrophysiological properties of thalamic GABAergic transmission. Miniature inhibitory postsynaptic currents (mIPSCs), resulting from spontaneous GABA vesicle release, were recorded for 3–5 min at a −70 mV holding potential, with NMDA and AMPA receptors blocked by AP5 (50 μM) and CNQX (25 μM), respectively (Fig. 2A). No significant differences were observed between control-1D and control-7D rats (amplitudes: 23.01 ± 3.62 pA *vs.* 25.30 ± 3.56 pA; frequencies: 0.89 ± 0.27 Hz *vs.* 0.73 ± 0.19 Hz; p > 0.05) and the data were pooled into the control group. Compared with the control or CFA-1D groups, mIPSCs in the CFA-7D group showed similar amplitudes (Fig. 3B, control: 24.23 ± 2.48 pA; CFA-1D: 21.57 ± 2.83 pA; CFA-7D: 22.04 ± 1.96 pA; *F*_2,50_ = 0.333, p = 0.718), but significantly higher frequencies [Fig. 3C, control: 0.69 ± 0.12 Hz; CFA-1D: 0.91 ± 0.16 Hz; CFA-7D: 1.18 ± 0.12 Hz, F(2, 50) = 3.586, p = 0.035]. These changes indicate an increased probability of spontaneous GABA release in chronic inflammatory pain rats^17^.

We next recorded the paired-pulse ratios (PPRs) of IPSCs, which inversely correlated with activity-driven presynaptic GABA release^18^,^19^,^20^. Figure 3D showed representative IPSCs evoked by paired-pulse stimulations with intervals of 50 ms. PPRs significantly increased on day 7 after CFA injection [Fig. 3E, F(1, 49) = 24.46, p < 0.001, at 25-ms interval: 0.69 ± 0.21 *vs.* 1.90 ± 0.62, p < 0.05; at 50-ms interval: 0.69 ± 0.22 *vs.* 1.65 ± 0.40, p < 0.05; at 100-ms interval: 0.56 ± 0.15 *vs*. CFA-7D 1.67 ± 0.28, p < 0.01. at 200-ms interval: 0.67 ± 0.20 *vs.* 1.18 ± 0.47, p > 0.05]. These results indicate a deceased activity-driven GABA release capacity derived from TRN neurons in chronic inflammatory pain rats.

**Figure 4:**
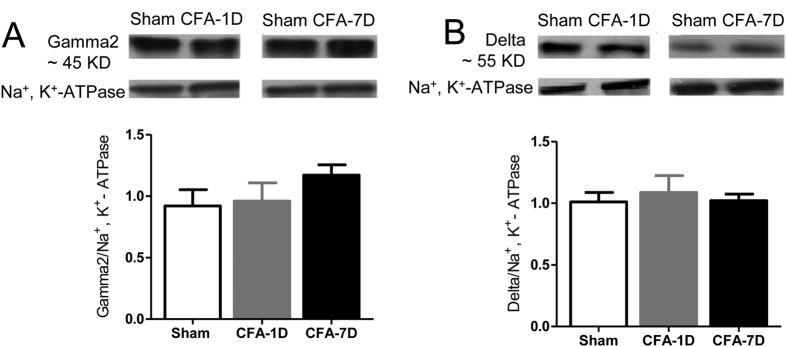
Lack of significant changes in the expression of GABA_A_R gamma2 and delta subunits in the VB of inflammatory pain rats. We did no detect significant changes in protein expression of the GABA_A_R gamma2 subunit ((**A**) ~45 KD) or delta subunit ((**B**) ~55 KD) in CFA-induced inflammatory pain rats. Na^+^, K^+^-ATPase was used as the reference in the Western blotting. Control: normal saline injection into hindpaws of mice. CFA-1D or CFA-7D: day 1 or day 7 after CFA injection to hindpaws of mice. Error bars indicated SEMs, unpaired t test.

**Figure 5:**
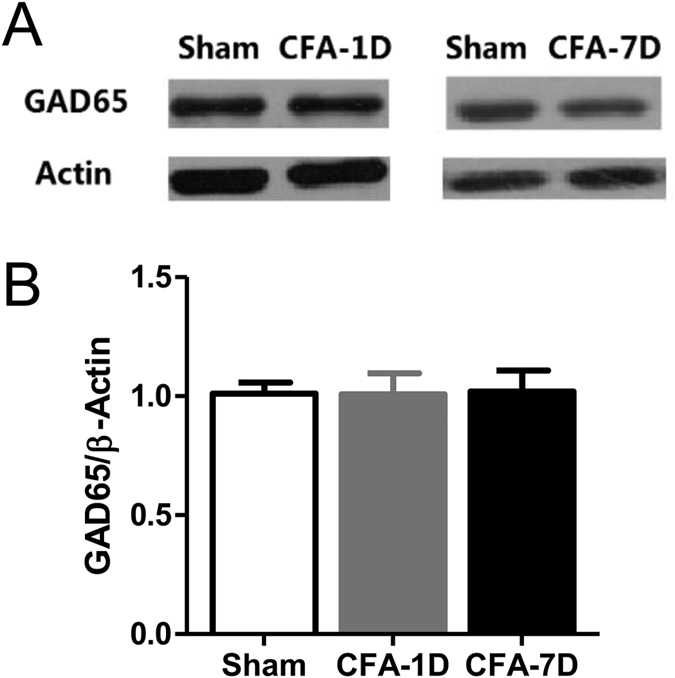
Lack of significant changes in the expression of GAD65 in the VB of inflammatory pain rats. (**A**) Representative Western blotting bands of GAD65 (~65 KD). β-actin was used as the reference. (**B**) Statistics of relative quantification of GAD65 in the control, CFA-1D (day 1 after CFA injection) and CFA-7D (day 7 after CFA injection) groups. Error bars indicated SEMs, unpaired t test.

Changes in both mIPSC frequencies and PPRs indicate a presynaptic origin of the altered GABAergic transmission^21^,^22^. Consistently, Western blotting did not reveal significant changes of protein expression of gamma2 or delta subunits of GABA_A_Rs (Fig. 4 and Supplementary Fig. S1), or GAD65, a GABA synthesizing enzyme, in chronic inflammatory pain rats (Fig. 5 and Supplementary Fig. S2).

Overall, the above results indicate altered GABAergic transmission in the VB in rats with chronic inflammatory pain. However, it is not known whether these changes exacerbate or alleviate chronic inflammatory pain. We next performed a series of pharmacological, transgenic and optogenetic manipulations to rescue or simulate the changes in thalamic GABAergic transmission, and examined their effects on nociceptive behaviors.

### Activation of GABA_A_Rs with muscimol in the VB: facilitation of physiological and acute inflammatory pain but attenuation of chronic inflammatory pain

**Figure 6:**
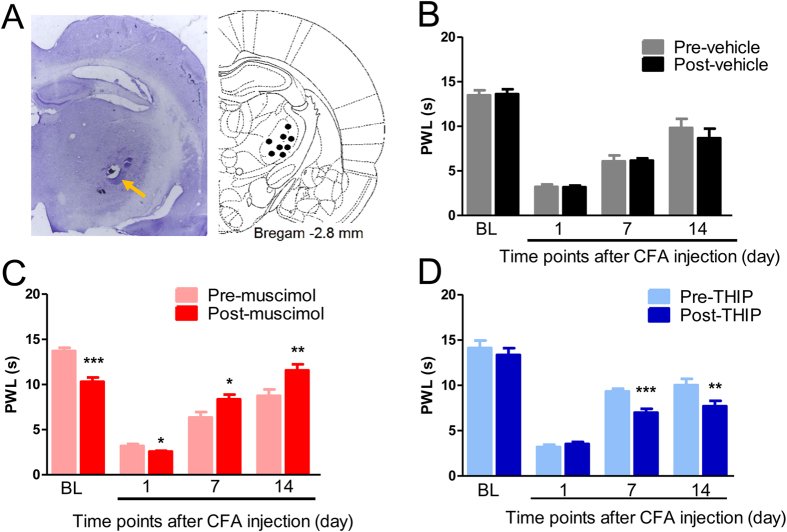
Effects of VB injection of muscimol and THIP on paw withdrawal latencies (PWLs). Histological illustration of microinjection sites at Bregma −2.8 mm. (**B**) Vehicle (normal saline) injection did not affect paw withdrawal latencies (PWLs). (**C**) Muscimol decreased PWLs in naïve and acute inflammatory pain rats (CFA-1D), but increased PWLs in chronic inflammatory pain rats (CFA-7D and CFA-14D). (**D**) Activation of extra-synaptic GABA_A_Rs with THIP had limited effects on PWLs in naïve or acute inflammatory pain rats (CFA-1D), but significantly attenuated thermal hyperalgesia in chronic inflammatory pain rats (CFA-7D and 14D). Error bars indicated SEMs, n = 10–12, *P < 0.05, **P < 0.01, ***P < 0.001, paired t tests.

We first examined the effects of intra-VB injection of the GABA_A_R agonist muscimol on thermal nociceptive thresholds in normal and inflammatory rats (Fig. 6A). As a control, intra-VB injection of vehicle (normal saline) did not affect the paw withdrawal latency (PWL) in naïve (Fig. 6B, 13.47 ± 0.57 s *vs.* 13.61 ± 0.54 s, p = 0.889) or inflammatory rats (CFA-7D: 6.31 ± 0.78 s *vs.* 6.10 ± 0.38 s, p = 0.449; CFA-14D: 9.42 ± 0.99 s *vs.* 8.14 ± 0.54 s, p = 0.667). Intra-VB injection of muscimol significantly reduced PWLs in naïve (13.71 ± 0.35 s *vs.* 10.29 ± 0.48 s, p < 0.001) and acute inflammatory pain rats (CFA-1D: 3.2 ± 0.21 s *vs.* 2.57 ± 0.08 s, p = 0.028), indicating an algesic effect of thalamic GABAergic transmission under physiological and acute inflammatory pain conditions. By contrast, intra-VB injection of muscimol produced significant relief of the thermal hyperalgesia in CFA-induced chronic inflammatory pain rats (CFA-7D: 6.35 ± 0.59 s *vs.* 8.35 ± 0.53 s, p = 0.043; CFA-14D: 8.74 ± 0.70 s *vs.* 11.56 ± 0.66 s, p = 0.003).

These results indicate dual effects of thalamic GABA_A_R activation on nociceptive behaviors: it facilitates physiological and acute inflammatory pain, but attenuates thermal hyperalgesia in chronic inflammatory pain.

### Activation of extra-synaptic GABA_A_Rs with THIP: facilitation on chronic inflammatory pain

Muscimol activates both synaptic and extra-synaptic GABA_A_Rs, which have distinct electrophysiological dynamics in controlling neuronal activities^23^. THIP is a preferential agonist for delta subunits, the most abundantly expressed extra-synaptic subunit of GABA_A_Rs in the VB^24^,^25^,^26^. Intra-VB injection of THIP did not affect PWLs in naïve (14.11 ± 0.83 s *vs.* 13.35 ± 0.76 s, p = 0.504) or acute inflammatory pain rats (CFA-1D: 3.19 ± 0.26 s *vs.* 3.53 ± 022 s, p = 0.132), but significantly reduced PWLs in chronic inflammatory pain rats (CFA-7D: 9.32 ± 0.30 s *vs.* 6.98 ± 0.43 s, p = 0.0003; CFA-14D: 10.01 ± 0.70 s *vs.* 7.69 ± 0.61 s, p = 0.0037, Fig. 6D). These results suggest that selective activation of extra-synaptic GABA_A_Rs with THIP facilitates thermal hyperalgesia in CFA-induced chronic inflammatory pain.

Overall, the different effects of muscimol and THIP injections indicate distinct roles of synaptic and ambient thalamic GABA in thermal hyperalgesia in chronic inflammatory pain, with the former exerting analgesic and the latter algesic effects.

### Optogenetic activation of GABAergic transmission in the VB: attenuation of CFA-induced chronic inflammatory pain

**Figure 7:**
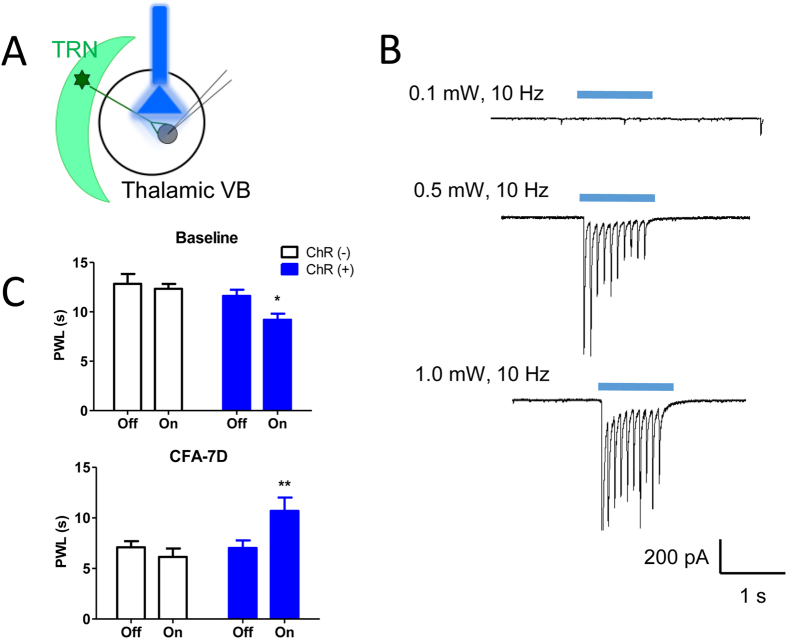
Optogenetic activation of GABAergic transmission in the VB attenuated CFA-induced thermal hyperalgesia. (**A**) Experimental configuration showing the location of optical fibers. Light was delivered to ChR2-expressing TRN terminals in the VB of VGAT-ChR2 mice with evoked IPSCs recorded from a thalamic relay neuron. (**B**) *In vitro* whole-cell patch clamp recordings of evoked IPSCs with 10 Hz, 465 nm blue light. IPSCs were successfully evoked with 0.5 mW and 1.0 mW stimulation and inhibited by 25 μm gabazine. (**C**) Activation of contralateral thalamic GABAergic neurons with 465 nm blue light stimulation decreased paw withdrawal latencies (PWLs) in naïve mice but attenuated CFA-induced thermal hyperalgesia in VGAT-ChR2 mice (ChR^+^). Photo-stimuli did not affect mice without ChR2 protein (ChR^−^). Error bars indicated SEMs, *P < 0.05, **P < 0.01, paired t test. ChR: Channelrhodopsin-2; Off: light off; On: light on.

The currents of synaptic GABA_A_Rs are mainly mediated by presynaptic GABA release from vesicles. We next applied optogenetics to examine the hypothesized protective role of synaptic GABAergic transmission in chronic inflammatory pain. The majority of GABAergic innervations to the VB come from the TRN in rodents^5^. To mimic the phasic inhibition mediated by synaptic GABA_A_Rs, optical fibers were implanted in the VB to activate ChR2-expressing TRN terminals in VGAT-ChR2-EYFP transgenic mice (Fig. 7A). Effective illumination parameters were first determined using *in vitro* whole-cell patch-clamp recordings. 20 ms pulses of a 465 nm blue light with an intensity of 0.5 or 1.0 mW induced IPSCs (Fig. 7B).

Previous studies have shown a 10–20 Hz spontaneous discharge rate of TRN neurons in freely-moving rodents^27^,^28^. IPSCs were effectively evoked by 10 Hz trains (Fig. 7B). At 20 Hz and 50 Hz, however, IPSCs could only be evoked by the first couple of photo-stimuli, but not the following stimuli (data not shown), possibly due to the desensitization of ChR2 currents^29^. An activation power of 3.0 mW could activate a much broader VB response^30^, prompting us to observe nociceptive behaviors of VGAT-ChR2 mice under 10 Hz and 3.0 mW blue light (465 nm) stimulations in the following experiments.

Activation of contralateral thalamic TRN terminals in the VB facilitated nociceptive behaviors in naïve mice (Fig. 7C, baseline, ChR2(−): 12.81 ± 0.77 s *vs.* 12.81 ± 0.52 s, p = 0.970; ChR2(+): 11.62 ± 0.62 s *vs.* 9.21 ± 0.61 s, p = 0.028, light off *vs.* light on), similar to the data of muscimol injection. In inflammatory mice, by contrast, activation of contralateral thalamic TRN terminals attenuated the thermal hyperalgesia (CFA-7D, ChR2(−): 6.99 ± 1.24 s *vs.* 7.99 ± 3.06 s, p = 0.354; ChR2(+): 6.24 ± 1.37 s *vs.* 10.77 ± 1.80 s, p = 0.003). These data confirm the analgesic effects of synaptic GABAergic transmission in the VB in chronic inflammatory pain.

### Selective knockout of synaptic GABA_A_R gamma2 subunits in the VB: delay in the recovery of CFA-induced chronic inflammatory pain

**Figure 8:**
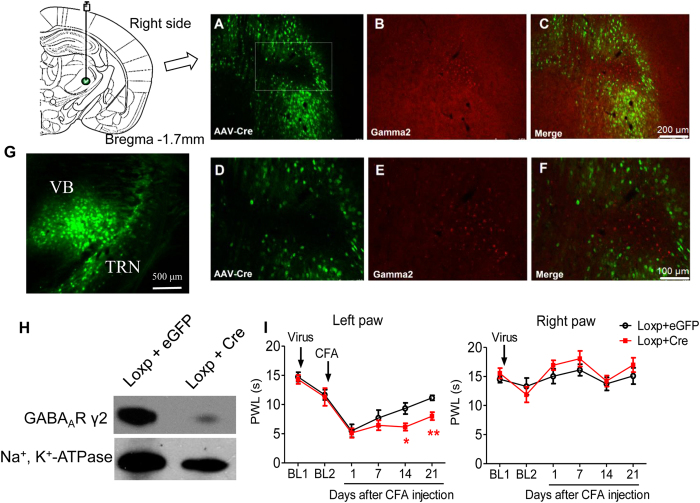
Selective knockout of synaptic GABA_A_R gamma2 subunits in the VB delayed the recovery of CFA-induced chronic inflammatory pain. (**A**–**C**) GABA_A_R gamma2 subunit knockout using the Cre-LoxP system. Immunofluorescent double staining of GABA_A_R gamma2 subunits (red) with AAV-Cre vector (green) into the VB of Gabrg2^*tm2Lusc*^/J mice. Gamma2 subunits were knocked-out in AAV-Cre-transfected thalamic relay cells. (**D**) and (**E**) Localization and expression of AAV-Cre/AAV-eGFP in the right VB at Bregma −1.7 mm. (**F**) GABA_A_R gamma2 subunit expression in AAV-Cre/AAV-eGFP-injected Gabrg2^*tm2Lusc*^/J mice with Western blot. The protein level of gamma2 subunits significantly decreased in AAV-Cre-injected, but not AAV-eGFP-injected mice. (**G**) Thalamic GABA_A_R gamma2 subunit knockout delayed the recovery from CFA-induced inflammatory pain (Left paw, CFA-14D and CFA-21D) without affecting contralateral PWLs (**H**) or acute inflammatory pain (CFA-1D). Error bars indicated SEMs, *P < 0.05, **P < 0.01, two-way ANOVA with Bonferroni post-test. PWL: paw withdrawal latency.

The above findings led to the assumption that inhibiting synaptic GABAergic transmission would delay the recovery of inflammatory pain. To test this hypothesis, we examined CFA-induced inflammatory pain in mice with selective knockout of VB GABA_A_R gamma2 subunits, the most abundant synaptic subunits in the thalamus^7^,^9^. Immunofluorescence staining and Western blotting confirmed the knockout of GABA_A_R gamma2 subunits in the VB by injection of AAV-Cre virus into Gabrg2^*tm2Lusc*^/J mice (Fig. 8A–F).

Gamma2 subunit knockout had limited effects on the nociceptive thermal threshold in naïve or acute inflammatory mice (Fig. 8G). In contrast, gamma2 subunit knockout significantly delayed the recovery from chronic inflammatory pain [Fig. 8G, 14 day: 9.34 ± 0.96 s *vs.* 6.15 ± 0.64 s, F(4, 72) = 2.89, p = 0.028; 21 day: 11.14 ± 0.43 s *vs*. 8.01 ± 0.68 s, F(4, 72) = 3.65, p < 0.01] during the whole process. These results indicate a protective role of synaptic GABAergic transmission on thermal hyperalgesia in chronic inflammatory pain.

## Discussion

Pain pathways share a common projection in the somatosensory thalamic nuclei (VPL/VPM). While the VPM is the area representing the body, both VPL and VPM are subject to GABAergic modulation by conditioning stimulation of hindlimb afferents^4^. The majority of GABAergic afferents of the VB come from thalamic TRN in rodents, with a small part form the zona incerta and local interneurons^5^. The TRN is a reservoir of GABAergic interneurons and concerns with most, if not all, functional modalities resulted from its complexity^31^. The reduction in extracellular GABA levels (Fig. 1) and concomitant decreased tonic inhibition (Fig. 2) thus most possibly result from decreased GABA release from the presynaptic TRN.

We also detected increased frequencies of mIPSCs and increased PPRs (Fig. 3). These two measurements both reflect presynaptic functions but are distinct in their mechanisms: mIPSCs result from spontaneous GABA release from presynaptic vesicles, whereas PPRs reflect release capacity of GABA evoked by activities (action potentials). Spontaneous and activity-dependent releases originate from distinct vesicle pools with limited cross-talk between each other^32^,^33^. In addition, the highly regulated nature of evoked neurotransmitter release is in sharp contrast to spontaneous vesicle fusion, which is only loosely regulated by extracellular or intracellular calcium^34^. Knockout of synaptotagmin 1 or complexins attenuates evoked synchronous neurotransmitter release without affecting spontaneous release^35^, whereas deletion of synaptobrevin-2/VAMP2 reduces spontaneous fusion rate^36^. Finally, vesicles in the spontaneously release pool are more likely to refuse spontaneously compared to vesicles released with activity, enabling an increased frequency^32^. Increased PPRs indicate decreased GABA release capacity driven by activities, consistent with decreased extracellular GABA levels and the fact that repeated noxious stimuli depress neuronal firing in the TRN^37^. By contrast, spontaneously release events, as measured with mIPSCs, are closely associated with synaptic development and plasticity, including stabilization of synaptic networks^38^,^39^. Thus, neuroplastic changes of spontaneous releases may occur in chronic inflammatory pain, which give rise to structural and/or functional alterations of the TRN-VB circuit.

These results support several previous reports. Partial peripheral nerve injury promotes a loss of GABAergic inhibition in the dorsal horn^21^, whereas neuroimaging studies show a significant reduction of gray matter volumes and blood flow of the TRN in patients with chronic pain^13^,^40^. Another recent study in chronic neuropathic pain reports increased oscillatory activities in the thalamus were associated with altered neuron-astrocyte couplings^41^. Altered GABA signaling by astrocytes leads to local circuit imbalance in some pathological conditions, such as Alzheimer’s disease^42^ and epilepsy^43^. Similar mechanisms may also contribute to chronic pain. This hypothesis is also consistent with stronger nociceptive perception in females than males: synaptic GABA_A_Rs subunits in the VB are lower in the female^44^. In addition, GABAergic transmission in the VB could be increased by the high levels of plasma estradiol during the preestrous and estrous phases, which are associated with attenuated pain in the estrous cycle^45^. With various manipulations, we show that manipulating thalamic GABAergic transmission significantly affects thermal hyperalgesia in chronic inflammatory pain.

In addition to these reflex behaviors, the VB has also been implicated in spontaneous pain behaviors, such as conditioned place preference^46^. However, we did not include this paradigm in the present study. In addition, although evoked activities of thalamic neurons are preferentially mediated by GABA_A_Rs^47^, some studies have also implicated a significant role of GABA_B_Rs^48^,^49^. GABA_A_Rs and GABA_B_Rs could interact with each other though different mechanisms^47^. Decreased mRNA expression of GABA_B_R1/2 subunits has been reported in the thalamus of chronic monoarthritic animals^48^,^50^. But the exact mechanisms of GABA_B_Rs’ involvement in chronic pain require further investigation.

Thalamic GABAergic transmission plays different roles in thermal hyperalgesia under acute and chronic inflammatory conditions. One interesting finding in the present study is the distinct effects of various manipulations on physiological or acute thermal pain versus chronic thermal hyperalgesia^22^. VB injection of muscimol or photo-stimulation of the TRN-VB GABAergic pathway significantly facilitated physiological thermal pain or acute thermal hyperalgesia but inhibited chronic thermal hyperalgesia (Figs 6C and 7C). Similar biphasic effects, which have also been reported in the primary somatosensory cortex^51^, most likely result from distinct neuronal effects of GABA_A_ receptor activation^52^, depending on the basal tone of GABAergic transmission and the sensitivity of GABA_A_Rs under different conditions^53^. The polarity of GABAergic currents critically depends on the intracellular chloride concentration and the expression of Na^+^-K^+^-2Cl^−^ cotransporters (NKCC1) and K^+^-Cl^−^ cotransporters (KCC2)^54^. High expression of NKCC1 and low (or no) expression of KCC2 keep a high intracellular Cl^−^ concentration, which induces depolarization or excitatory responses to GABA. Recent studies indicate that peripheral nerve injury-induced neuropathic pain is associated with decreased KCC2 levels in the thalamus, especially in the chronic phase^15^. Similar changes might happen in chronic inflammatory pain, which would give a possible interpretation of the opposite effects of optogenetics in acute and chronic pain. In addition to intracellular Cl^−^ concentrations, the excitatory or inhibitory effect of GABA is also affected by the biphasic change in GABA_A_ conductance and shunting inhibition in TRN interneurons^52^.

One direct way to elucidate the distinct effects of thalamic GABAergic transmission on acute and chronic pain would be *in vivo* recording of thalamic neurons. Thalamic reticular cells demonstrate electrophysiological diversities: burst firing mode in slow-wave sleep, single spike mode or tonic activities during the waking state^28^, and more complex firing mode under anaesthesia^55^. Our previous studies and many others have found that chronic pain is accompanied by altered EEG^56^,^57^ or thalamocortical dysrhythmia, where the TRN plays a leading role in the generation of pathological oscillations^58^. In the present study, optogenetic activation of contralateral thalamic TRN GABAergic interneurons or pharmacological activation of GABA_A_Rs with muscimol attenuated CFA-induced chronic inflammatory pain, confirming this hypothesis.

For thalamic relay cells, two main firing modes have been reported: tonic and burst firing. Activation of extra-synaptic GABA_A_Rs hyperpolarizes neurons and promotes burst firing, whereas blockage of these receptors promotes tonic-over-burst firing^1^. However, conflicting evidence is present regarding the relationship between these firing modes and pain behaviors. Several studies correlate the tonic firing of thalamic relay cells to nociceptive pain, and the burst firing to inhibition on nociceptive information processing^12^,^59^. Mice with T-type calcium channel knockout, for example, show depleted burst firing in the thalamus and stronger visceral pain responses^59^. By contrast, positive or lack of correlations between burst firing in the sensory thalamus with pain symptoms have been reported by other studies^1^,^60^,^61^,^62^. Finally, some researchers suggest that only specific bursting firing in the VB has the anti-nociceptive effect^63^,^64^.

Another complexity of findings from the present study lies in the distinct effects of synaptic and ambient GABA, which binds to synaptic and extra-synaptic GABA_A_Rs different in their molecular heterogeneity and effects on neuronal excitability. Unlike the dual effects of phasic GABAergic transmission between low and high GABA levels, tonic GABAergic inhibition positively and dose-dependently correlates with the levels of GABA^65^. In the present study, a decreased tonic inhibition in the VB was observed in chronic inflammatory pain rats (Fig. 2B). Optogenetic activation of the TRN-VB GABAergic pathway produced similar analgesic effects to muscimol injection, which was more sensitive to synaptic than extra-synaptic GABA_A_Rs. This conclusion was further confirmed with knockout of γ2 subunits, the most abundant synaptic GABA_A_R subunit in the thalamus. Injection of THIP, a preferential agonist of the extra-synaptic δ subunit, yielded an opposite, algesic effect in chronic inflammatory pain. These findings suggest that reduced synaptic GABAergic transmission in the VB contributes to thermal hyperalgesia in chronic inflammatory pain, whereas the reduction of tonic inhibition of extra-synaptic GABA_A_Rs in the VB might play a protective role. However, it needs to be noted that the distinction between synaptic and extra-synaptic GABAergic transmission could not be dissociated unequivocally. *In vivo* recording would be required to more directly correlate synaptic and ambient GABAergic transmission with neuronal firing modes and pain behaviors.

## Conclusions

In conclusion, the present study supports a hypothesis that chronic inflammatory pain is associated with reduced GABAergic transmission in the VB, which contributes to persistent thermal hyperalgesia. Therapeutic interventions targeting GABAergic transmission in the VB might benefit the relief from chronic inflammatory pain.

## Materials and Methods

### Animals

Male Sprague-Dawley rats were provided by the Department of Experimental Animal Sciences, Peking University Health Science Center. VGAT-ChR2-EYFP line 8 BAC transgenic mice (https://www.jax.org/strain/014548) and Gabrg2^*tm2Lusc*^/J mice (https://www.jax.org/strain/016830) were purchased from Jackson Laboratories. Animals were housed under a 12-hour alternating light/dark cycle with food and water available *ad libitum*, and handled before behavioral experiments. All experimental procedures were conducted in accordance with the guidelines of the International Association for the Study of Pain and were approved by the Animal Care and Use Committee of Peking University.

### CFA model of chronic inflammatory pain and behavioral testing

CFA (100 μl for rats of ~300 g or 50 μl for mice of ~8 weeks, Sigma-Aldrich) was intraplantarly injected into the left hind paw to induce inflammatory pain^66^. The same volume of normal saline (NS) was injected into the left hind paw as the control group. Paw withdrawal latencies (PWLs) were measured with 40 W (for rats) or 15 W (for mice) radiant light focused on the left plantar surface. A 30 s (for rats) or 20 s (for mice) cut-off time was applied to avoid unnecessary tissue damage. Animals were adapted to the test environment for at least 20 min before tests. PWLs were measured before (as the baseline), and 1, 7, and 14 days after CFA injection. The tests were repeated 3 times with 3–5 min intervals.

### Microdialysis analysis of GABA in the VB

Microdialysis of extracellular levels of GABA was performed in rat VB, and GABA in the perfusates was analyzed with high performance liquid chromatography (HPLC) coupled with electrochemical detector (ECD), as described previously^67^. In brief, the guiding cannula (CMA/11, Harvard Apparatus, Holliston, MA) was implanted into the right VB according the following coordinates: anterior–posterior (AP) 3.6 mm; medial–lateral (ML) 3.6 mm; and dorsal–ventral (DV) 6.5 mm relative to the Bregma. Four or five skull screws were used for securing the guiding cannula to the skull surface with dental acrylic. Before sampling, probes (CMA/11, Harvard Apparatus, Holliston, MA) with 2-mm cuprophane membranes were inserted carefully into contralateral VB overnight for stabilization with a very slow perfusion rate (0.1 μl/min). Previous studies had shown good efficiency (>90%) for detection of neurotransmitters at the 0.1 μl/min perfusion rate^29^,^68^,^69^. O-phthalaldehyde (OPA, Sigma-Aldrich) with sodium sulfite (Sigma-Aldrich) was chosen as the derivate reagent^70^. Standards were obtained from Sinopharm Chemical Reagent.

### Thalamocortical slice preparation and whole-cell patch-clamp recordings

Male Sprague-Dawley rats, weight 200 ± 20 g, were anesthetized with isoflurane (Shenzhen RWD Life Science Co., Ltd.) and perfused transcardially with chilled sucrose-based cutting solution (in mM): 234 sucrose, 2.5 KCl, 1.25 NaH_2_PO_4_, 25 NaHCO_3_, 0.5 CaCl_2_, 7 MgSO_4_ and 10 glucose (pH 7.4; 335–340 mOsm). The brain was removed quickly, and somatosensory thalamocortical slices^71^ were cut at 350 μm with a vibratome (VT1000S, Leica Geosystems, Heidelburg, Germany). These slices were incubated in 37 °C artificial cerebral spinal fluid (aCSF) solution (in mM) consisting of 125 NaCl, 2.5 KCl, 1.25 NaH_2_PO_4_, 25 NaHCO_3_, 2 CaCl_2_, 2 MgCl_2_ and 10 glucose (305–310 mOsm), aerated with 95% O_2_ and 5% CO_2_ to a final pH of 7.40. After 1 h incubation, slices were transferred carefully to the recording chamber by super-fusing aCSF at the room temperature.

A Multiclamp 700B amplifier and Clampex software (Molecular Device, Sunnyvale, CA) were used for electrophysiological recording. Thalamic relay cells were viewed using a 60× water-immersion lens with infrared differential interference contrast optics. Miniature inhibitory postsynaptic currents (mIPSCs) and tonic currents were recorded at a −70 mV holding potential. Pipettes (about 3–5 MΩ) were filled with an internal solution containing (in mM): 140 CsCl, 10 Hepes, 10 EGTA, 2 MgSO_4_, 1 CaCl_2_ and 2 Mg-ATP (pH 7.20–7.30, 290–300 mOsm). Data were filtered at 2 kHz and digitized at 10 kHz. D(−) 2-amino-5-phosphonovaleric acid (AP5, 50 μM), 6-cyano-7-nitroquinoxaline-2.3-dione sodium (CNQX, 20 μM), and tetrodotoxin (TTX, 0.5 μM) were used to block glutamatergic and sodium currents, respectively. All drugs used were purchased from Sigma-Aldrich except TTX (Institute for Aquatic Science and Technology, Qinhuangdao, China). The mIPSCs were analyzed with the Mini-Analysis software (version 6.0.7, Synaptosoft). Amplitudes of mIPSC thresholds were defined at 10 pA. Populations of mIPSCs were verified visually and the average amplitude and frequency were measured. Paired-pulse stimulations were given with 25, 50, 100 and 200 ms intervals using a monoelectrode. Tonic inhibition and paired-pulse ratios (PPRs) were analyzed using Clamp 10.1.

### Expression of GABA_A_R gamma2/delta subunits and GAD65

Contralateral VB from mice with the adeno-associated virus (AAV) vector expression (see below) or from rats 1 or 7 days after CFA injection were collected and immediately stored at −80 °C. Membrane proteins were extracted using the Membrane and Cytosol Protein Extraction Kit (Applygen Technologies Inc., Beijing). Equal amounts (15 μg) of membrane proteins of gamma2/delta subunits and total proteins of GAD65 were separated by SDS–PAGE on 10% gels and transferred to PVDF membranes (Merck Millipore). Membranes were blocked with 5% milk for 30 min at the room temperature and incubated at 4 °C overnight with rabbit anti-GABA_A_R primary antibodies (for the gamma2 subunit, 1:1000, Novus; for the delta subunit, 1:1000, Merck Millipore) and anti-GAD65 primary antibodies (1:5000, Abcam). The blots were then incubated with HRP-conjugated anti-rabbit secondary antibody (1:2000, Jackson, Bar Harbor, Maine). The bands were detected using an enhanced chemiluminescence detection kit (ECL, Santa Cruz Biotechnology, Inc.). Band intensity was quantified using the software Quantity One 4.6.2 (Bio-Rad Laboratories).

### Cannula implantation and microinjection of GABA_A_R agonists

Rats were anaesthetized deeply with 1% sodium pentobarbital (0.5 ml/100 g, *i.p*.) and placed in a stereotaxic frame. Guide cannulas (O.D. 0.56 mm/I.D. 0.38 mm, Shenzhen RWD Life Science Co., Ltd., China) were implanted bilaterally into the VB according to the following coordinates relative to the Bregma: AP −3.6 mm; ML −3.6 mm; and DV −6.5 mm. Four or five skull screws were used for securing the guide cannula to the skull surface with dental acrylic. Matching caps (Shenzhen RWD Life Science Co., Ltd., China) were inserted into guide cannulas and checked every day to prevent possible clogging. All animals were given at least one week for recovery before further experiments. The injection needle (Shenzhen RWD Life Science Co., Ltd., China), 0.5 mm lower than the guide cannula, was used for microinjection with a polyethylene catheter connecting a micro-syringe. GABA_A_R agonists muscimol (0.15 μg/μl, 0.5 μl, Tocris Bioscience) or THIP (4,5,6,7-tetrahydroisoxazolo(5, 4-c) pyridin-3-ol, 50 μM, 0.5 μl, Sigma-Aldrich)^51^,^72^ or vehicle (normal saline, 0.5 μl) were injected into the VB on either side over 2 min. The injection needle was held on for at least 1 min to allow for drug diffusion. Behavioral tests were performed 30 min after drug/vehicle injection. Rats with incorrect sites of the guide cannula were excluded from analysis and the final numbers of rats in each group were more than 10.

### Adeno-associated virus (AAV) vectors and knockout of the GABA_A_R gamma2

AAV-mCaMKII-Cre-eGFP (AAV-Cre) and AAV-mCaMKII-eGFP (AAV-eGFP) viruses were packaged and purchased from Sunbio Medical Biotechnology, Shanghai, China. Male Gabrg2^*tm2Lusc*^/J mice were microinjected in the VB (AP −1.6 mm; ML ±1.7 mm; and DV −3.5 mm) with 0.3 μl AAV-Cre or AAV-eGFP (as a control) at a speed of 0.1 μl/min after being anesthetized with 1% sodium pentobarbital (0.5 ml/100 g body weight, *i.p.*). The needle was kept on the site for 2 min to allow for virus diffusion and gradually withdrawn over 2 min to prevent possible leakage from the needle track. Two baseline measurements were performed: one before the injection of AAV virus and the other before CFA injection 3 weeks later. On days 1, 7, 14 and 21, PWLs were measured following the protocol described above. Only mice with correct and effective delivery of AAV virus into the VB were analyzed in behavioral tests.

### Immunohistochemistry for the GABA_A_R gamma2 subunit

Immunostaining and Western blotting (performed according to the protocol described above) were used to verify the knockout of AAV-Cre for GABA_A_R gamma2 subunits with Gabrg2^*tm2Lusc*^/J mice. For immunohistochemistry, thalamic sections (30-μm in thickness) were incubated with the primary antibody (a rabbit anti-GABA_A_R gamma2, 1:1000, Novus) in 0.5% BSA followed by Alexa Fluor 594-conjugated goat anti-rabbit IgG (1:200, Vitrogen Life Technologies) at 4 °C overnight. Sections were observed with a Leica DMI4000 microscope (Leica, Wetzlar).

### Optogenetics

Male VGAT-ChR2-EYFP line 8 (http://jaxmice.jax.org/strain/014548.html) mice (~8 weeks) were used in the optogenetic experiment. Following the same coordinates of the virus injection, the optical fiber (NA 0.37, O.D. 200 μm, Fiblaser Technology Co., Ltd, Shanghai, China) was implanted into the contralateral VB to drive the TRN terminals. Light-evoked IPSCs were recorded following the protocol of mIPSCs. A 465 nm blue light (PlexBright LED, Hong Kong Plexon) was delivered for evoked IPSCs at 0.1–3.0 mW with 10 Hz, 20 Hz or 50 Hz. In animal behavioral tests, in order to stimulate a larger area of the VB, 3.0 mW instead of 1.0 mW, 10 Hz, 465 nm blue light, was chosen to activate axonal terminals from the TRN. Based on predicted irradiance values measured in mammalian brain tissues, the light applied in the present study mainly activated neurons within an extent of no more than 1 mm (http://web.stanford.edu/group/dlab/cgi-bin/graph/chart.php), ensuring the specificity of the stimulation.

### Histology

After all experiments, animals were deeply anesthetized and perfused with 4% paraformaldehyde in phosphate buffer. Rats in the microdialysis and drug injection experiments received blue ink injection before perfusion to mark the site of sampling or injection. Thalamic sections (30-μm in thickness) were sliced coronally using a cryostat microtome to identify the tips of microdialysis probes, microinjection needles or optical fibers, according to The Rat/Mouse Brain Stereotaxic Coordinates^73^,^74^. Data from animals with incorrect localization excluded from analysis.

### Statistical analysis

All data were presented as means ± SEMs. Unpaired or paired student’s t tests were used for the comparisons of two groups. Data analysis of three groups was performed with one-way ANOVA with Tukey post-test. Comparisons of two groups with different time points were preformed using two-way ANOVA with Bonferroni post-test. The differences were calculated by the GraphPad Prism 5.0 and statistical significance was defined as p < 0.05.

## Additional Information

**How to cite this article**: Zhang, C. *et al*. Reduced GABAergic transmission in the ventrobasal thalamus contributes to thermal hyperalgesia in chronic inflammatory pain. *Sci. Rep.* **7**, 41439; doi: 10.1038/srep41439 (2017).

**Publisher's note:** Springer Nature remains neutral with regard to jurisdictional claims in published maps and institutional affiliations.

## Supplementary Material

Supplementary Information
