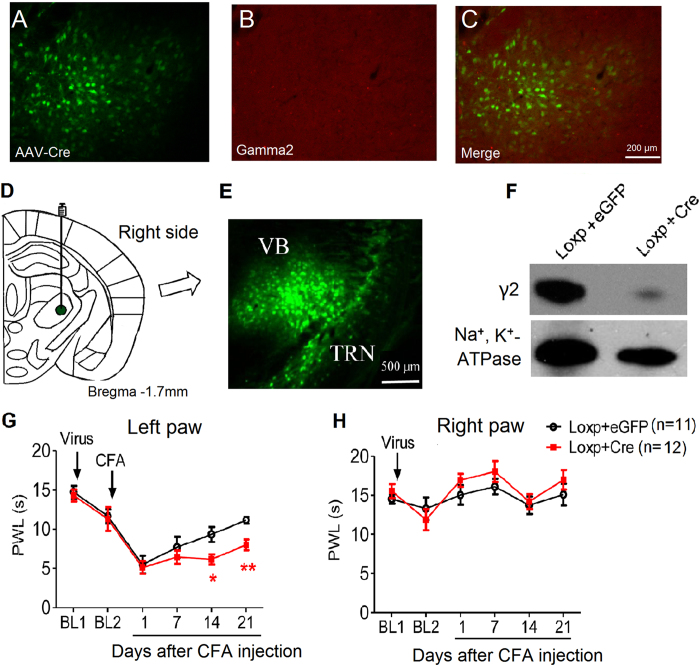# Erratum: Reduced GABAergic transmission in the ventrobasal thalamus contributes to thermal hyperalgesia in chronic inflammatory pain

**DOI:** 10.1038/srep46778

**Published:** 2017-05-04

**Authors:** Chan Zhang, Rong-Xiang Chen, Yu Zhang, Jie Wang, Feng-Yu Liu, Jie Cai, Fei-Fei Liao, Fu-Qiang Xu, Ming Yi, You Wan

Scientific Reports 7: Article number: 4143910.1038/srep41439; published online: 02 02 2017; updated: 05 04 2017

This Article contains errors in Figures 1, 2, 3, 4, 5, 6, 7 and 8. In Figure 1, n = 7 was omitted from panel C. In Figure 2, labels are missing from the bars of panel B. In Figure 3, ‘Control’ was incorrectly given as ‘Sham’, labels are missing from the bars in Figure 3B and C, and n = 7 and n = 9 were omitted from Figure 3E. In Figure 4, labels are missing from the bars. In Figure 5B, labels are missing from the bars. Figure 6A contains an incorrect panel. In Figure 6B,C and D, the x axes were incorrectly labelled as ‘Time points after CFA injection (day)’ and should read ‘Days after CFA injection’. In Figure 7C, n = 8 and n = 9 were omitted. The staining panels in Figure 8A–F were published in error. n = 11 and n = 12 were omitted from the ‘Right paw’ graph.

The correct figures appear below as Figures 1, 2, 3, 4, 5, 6, 7 and 8.

## Figures and Tables

**Figure 1:**
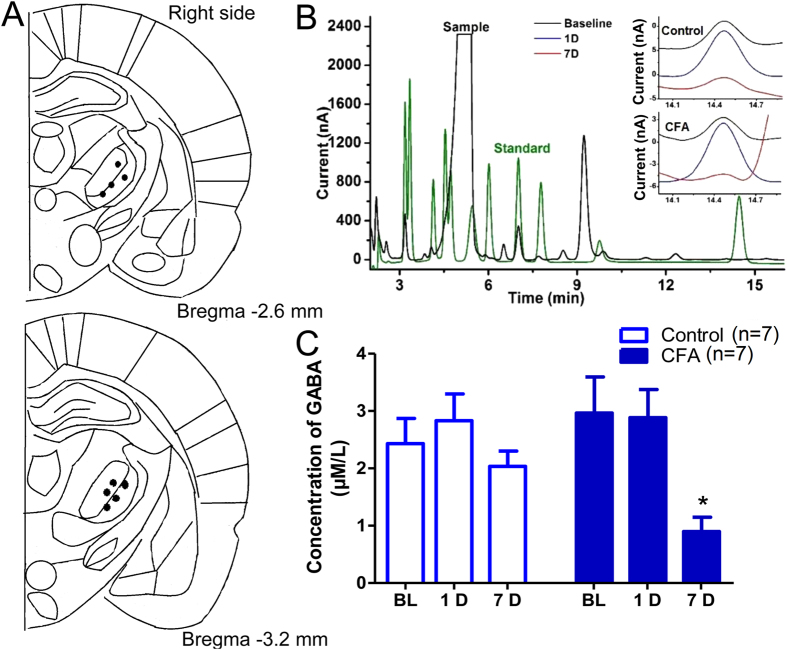


**Figure 2:**
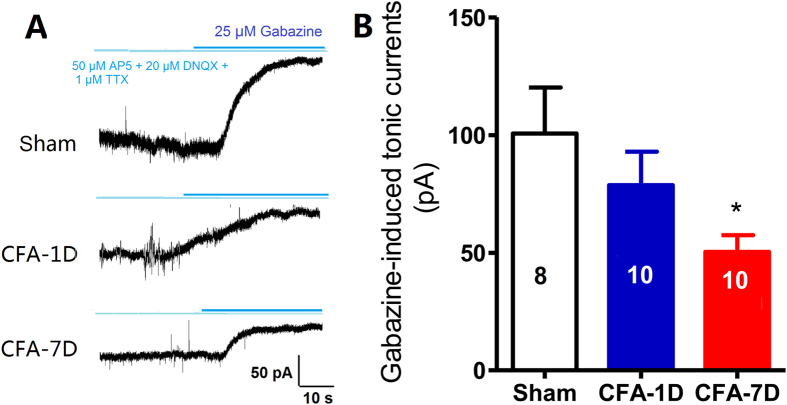


**Figure 3:**
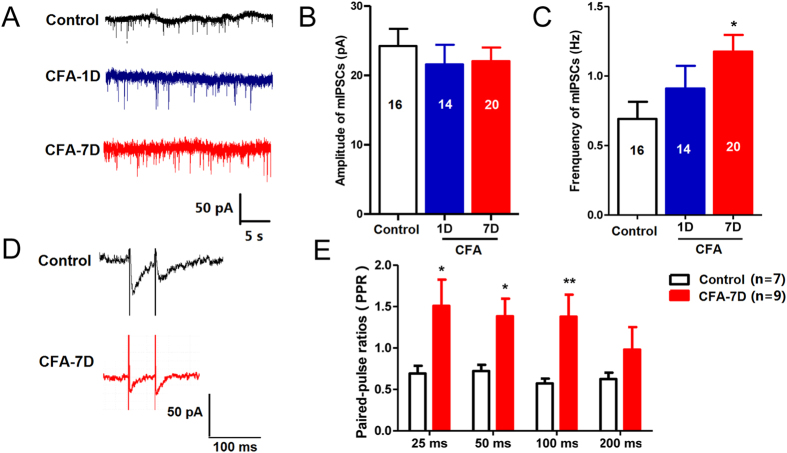


**Figure 4:**
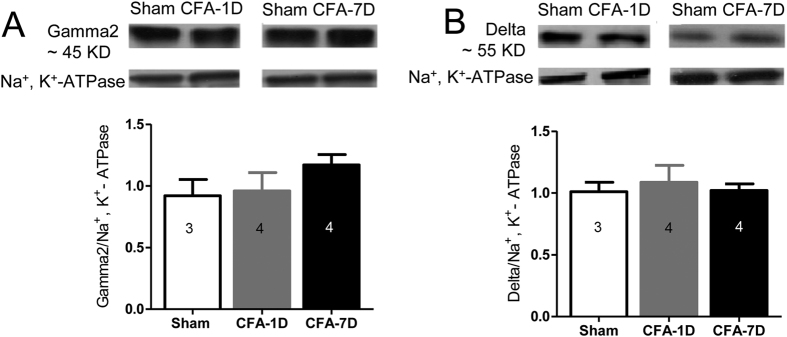


**Figure 5:**
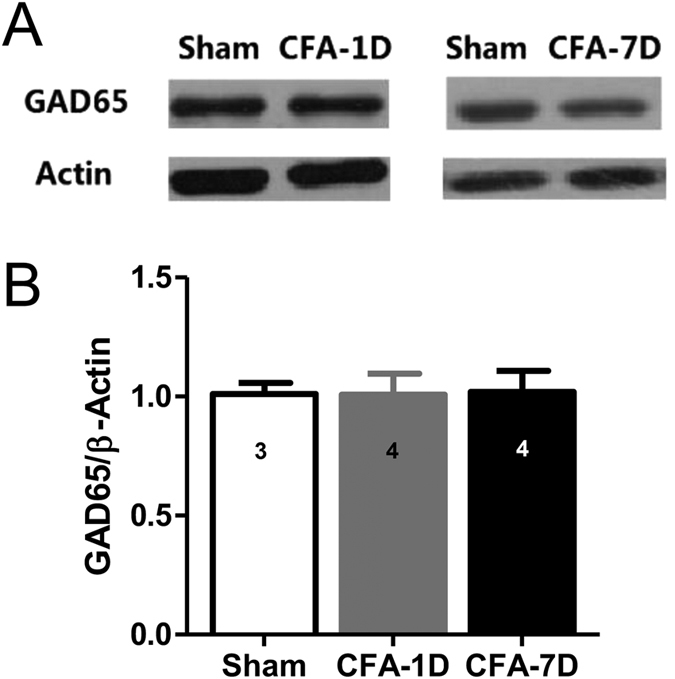


**Figure 6:**
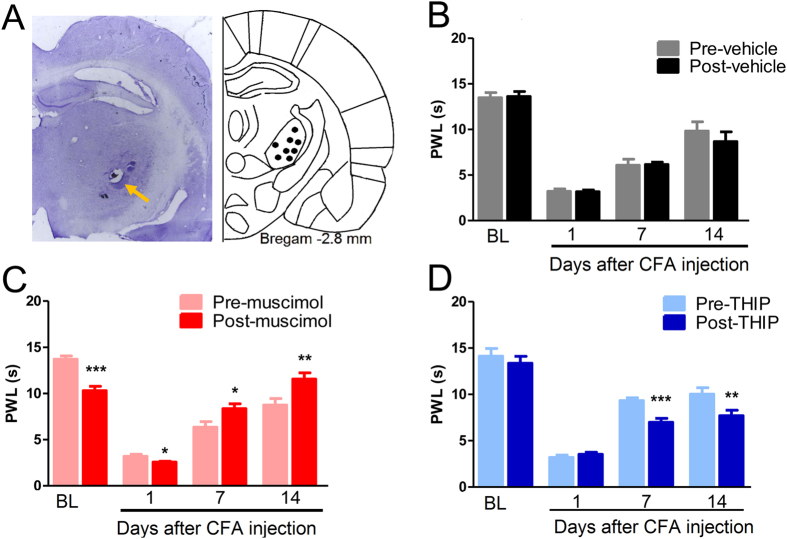


**Figure 7:**
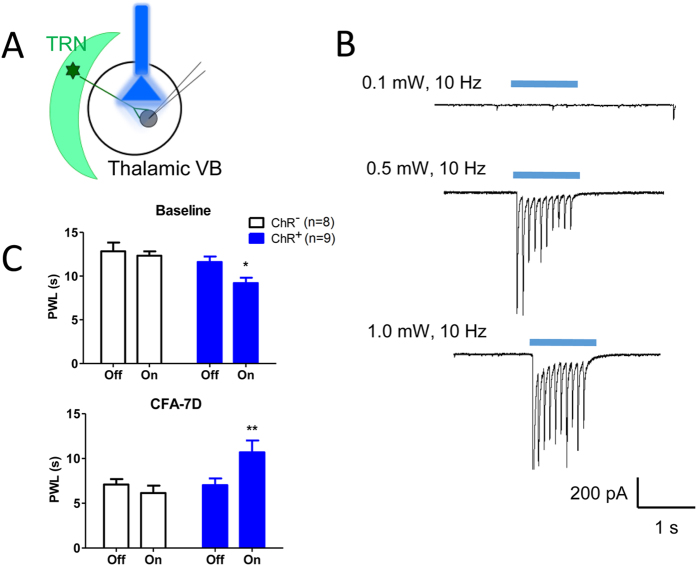


**Figure 8:**