# Supplementary material for: Reduced GABAergic transmission in the ventrobasal thalamus contributes to thermal hyperalgesia in chronic inflammatory pain
Source: Sci Rep. 2017 Feb 2;7:41439. doi: 10.1038/srep41439 (PMC5288727; doi:10.1038/srep41439)
Supplement: Supplementary Information [file srep41439-s1.pdf]

Supplementary Figures

Reduced GABAergic transmission in the ventrobasal thalamus contributes to thermal hyperalgesia in chronic inflammatory pain

Chan Zhang, Rong-Xiang Chen, Yu Zhang, Jie Wang, Feng-Yu Liu,Jie Cai, Fei-Fei Liao, Fu-Qiang Xu, Ming Yi, You Wan

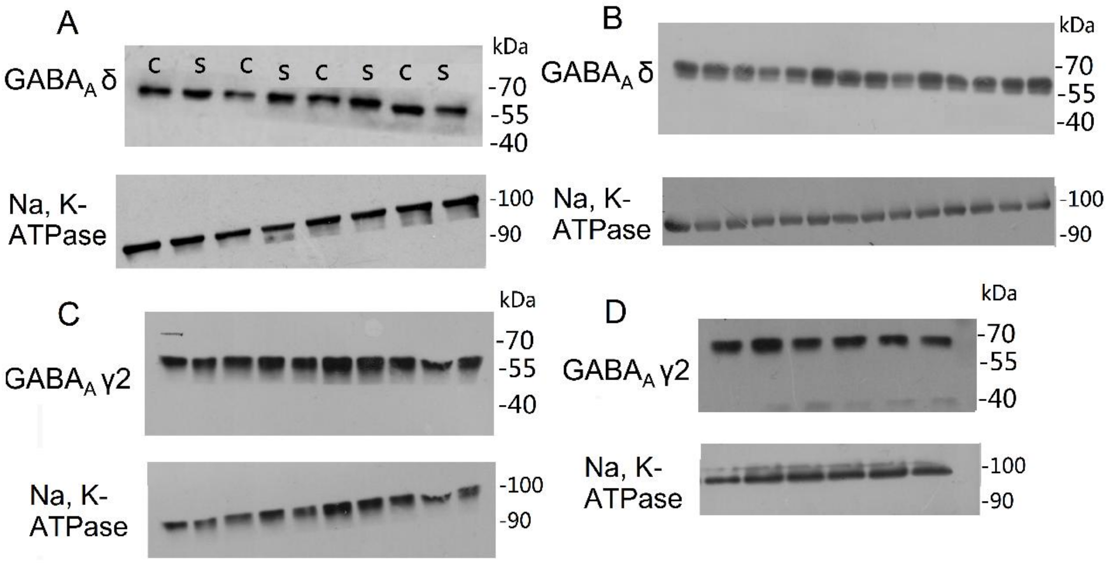

Supplementary Figure S1. Full-length Images of Western Blot of GABA$_A$R δ and γ2 Subunits Presented in Fig. 4

(A) and (B) Western blot of thalamic GABA$_A$R δ subunits in CFA-1D and CFA-7D. (C) and (D) Western blot of thalamic GABA$_A$R γ2 subunits in CFA-1D and CFA-7D groups. C: CFA group; S: Sham group.

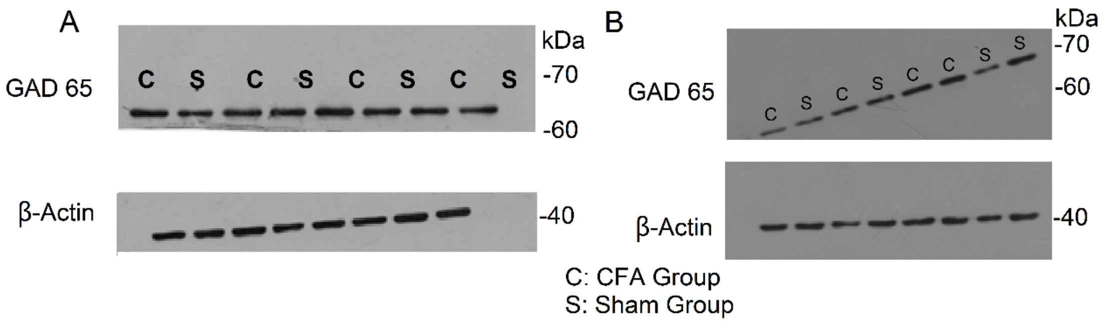

Supplementary Figure S2. Full-length Images of the Western Blot of GAD65 Presented in Fig. 5

(A) Western blot of thalamic GAD65 in acute pain (CFA-1D). (B) Western blot of thalamic GAD65 in chronic pain (CFA-7D). C: CFA group; S: Sham group.
